# Virucidal Efficacy of Holder Pasteurization of Breast Milk in a Portable Device

**DOI:** 10.3390/pathogens15091000

**Published:** 2026-09-21

**Authors:** Maxim I. Suchkov, Elizaveta V. Yakovchuk, Elena Y. Shustova, Dina I. Sirazova, Anastasia D. Zolotareva, Evgenia V. Karpova, Ekaterina L. Aksenova, Svetlana E. Sotskova, Liubov I. Kozlovskaya

**Affiliations:** 1 Chumakov Federal Scientific Center for Research-and-Development of Immune-and-Biological Preparations of RAS (Institute of Poliomyelitis), 108819 Moscow, Russia; suchkov_mi@mail.ru (M.I.S.); yakovchuk_ev@chumakovs.su (E.V.Y.); shustova_eu@chumakovs.su (E.Y.S.); sirazova_di@chumakovs.su (D.I.S.); zolotareva_ad@chumakovs.su (A.D.Z.); karpova_ev@chumakovs.su (E.V.K.); sotskova_se@chumakovs.su (S.E.S.); 2 Department of Organization and Technology of Production of Immunobiological Preparations, Institute for Translational Medicine and Biotechnology, Sechenov First Moscow State Medical University (Sechenov University), 119048 Moscow, Russia; 3 Biobank, Institute of Regenerative Medicine, Sechenov First Moscow State Medical University (Sechenov University), 119048 Moscow, Russia; ekaterina.aksenova.2001@mail.ru

**Keywords:** breast milk, virus, portable pasteurizer, virus inactivation, Holder pasteurization

## Abstract

Breast milk is the optimal nutrition for infants. However, a number of viruses can accumulate in breast milk and be transmitted to a newborn during breastfeeding. Holder pasteurization is a gold-standard method for breast milk treatment, achieving an acceptable compromise between microbiological safety and biological quality. We have evaluated the virucidal activity of Holder pasteurization of breast milk contaminated with RNA and DNA viruses in a portable home-use pasteurizer. Viruses were selected on basis of their ability to infect humans, contaminate breast milk, and pose a threat for a newborn. The efficacy was evaluated in experimental conditions using breast milk samples from four different donors. Infectious virus and viral genome content were assessed. Holder pasteurization resulted in a decrease in infectivity below the detection limit for enveloped RNA viruses (HIV-1, CHIKV, and SARS-CoV-2), but not RSV, and a partial decrease in infectious virus titer for non-enveloped RNA enteroviruses (EV-A71, E30, and PV1), and DNA viruses (hAdV5 and HSV-1). Moreover, infectivity reduction in E30 and PV1 in different samples of breast milk significantly differed. Therefore, Holder pasteurization can be a tool in the novel pandemic preparedness toolbox or probably help mothers manage breastfeeding during common infections.

## 1. Introduction

Breast milk rich in nutrients and bioactive components is a vital source of nutrition and immune protection for newborns, playing a key role in their growth, development, and disease prevention [1,2,3,4]. Moreover, in cases of newborn complicated medical histories (preterm birth, low and very low birth weight, complications, etc.), breast milk is considered a better nutrition option in comparison with formula [5].

Nevertheless, breastfeeding can serve as a route of mother-to-child transmission for certain viruses (Table 1): either via virus/virus-infected cell accumulation in breast milk from infected mothers or via virus transmission from a contaminated mother’s skin or blood. Risk of transmission depends on the virus and overall health of the nursing mother and infant [6,7,8]. For some infections there are guidelines, like the AAP ones [9], as to whether breastfeeding is recommended or should be avoided.

On the other hand, prematurely born, low and very low birth weight newborns require special feeding, but their increased susceptibility to infection poses the necessity of mitigation measures to reduce the risk of viral transmission through breast milk. Various methods have been used, including antiviral/antibacterial treatment of the breast milk, maternal antiviral therapy, and infant immunoprophylaxis.

Holder pasteurization (62.5 °C for 30 min) is recommended for donor human milk banks as a method possessing high antibacterial and antiviral activity while maintaining the nutritional and bioactive properties of breast milk [29,30].

Holder pasteurization effectively inactivates the infectivity of both free and cell-associated HIV-1, as well as HTLV, CMV, SARS-CoV-2, high-risk (types 16 and 18) and low-risk (type 6) HPV, Ebola, and Marburg viruses [31,32,33,34,35,36,37]. However, Holder pasteurization does not completely inactivate HBV in breast milk, making combined immunization and immunoglobulin injection at birth indispensable measures to prevent lactogenic transmission of the virus [38,39]. However, some data on the virucidal activity of the Holder pasteurization were obtained in different experimental settings and in very different matrices, not in milk [40], which could affect the results’ reliability.

In medical institutions and biobanks, pasteurization is carried out in big water bath pasteurizers (like PAS10000, HSC INOX, Décines-Charpieu, France). However, in some cases where a newborn has a complicated medical history or a mother has a common infection (herpesviruses, papillomaviruses, etc.), mothers and/or physicians consider the pasteurization of breast milk at home to protect a child instead of formula use. In such situations, portable pasteurizers with controlled specifications could be of help to create individual breast milk banks for each mother [41].

In the present study, we have evaluated the virucidal activity of Holder pasteurization of breast milk contaminated with a set of RNA and DNA viruses in a portable pasteurizer. Viruses (Table 2) were chosen as they can accumulate in the breast milk during acute infection of a mother (HIV-1, CHIKV, SARS-CoV-2, enteroviruses, and RSV) or can infect newborns during breastfeeding as well as contaminate milk during expression (HSV-1, hAdV5). Pasteurization efficacy was evaluated in experimental conditions using breast milk samples from four different donors. Infectious virus and viral genome content were assessed.

## 2. Materials and Methods

### 2.1. Breast Milk Specimens and Ethics Statement

Breast milk samples were received from the Biobank of the Sechenov University. Samples were collected from four healthy donors, aged 32–36, on their second or third pregnancies, breastfeeding for 1 to 6 months (Table S1), and stored frozen at −20 °C.

Breast milk donors signed an informed consent in Biobank for the use of their samples without personal information.

### 2.2. Cells and Viruses

The viruses and permissive cells used in the present work are summarized in Table 2. Their origins and passaging conditions are presented in the Supplementary Material.

### 2.3. Portable Pasteurizer

Study Portable pasteurizer for breast milk (SaveMyMilk LLC, Moscow, Russia), Russian Federation invention patent No. 2799703 dated 30 April 2023. It is a water bath system with an adjustable basket holder for two 150–300 mL bottles, with a pasteurization cycle compliant with Holder pasteurization: heating phase to 62.5 °C; holding time for 30 min with temperature controlled at 63.0 ± 0.5 °C.

### 2.4. Experimental Design

10^4.5^–10^6.5^ CCID_50_ of the virus was mixed with breast milk (1:9) in 1.5 mL test tubes (total volume of 200 µL) and submerged into a PET container with cow’s milk (approx. 290 mL). Then, the PET container was put in a bottle holder and placed in water in the portable pasteurizer, where the outside water level was above the cow’s milk level (Figure S1: The author generated this figure using Samsung AI, has reviewed and edited the generated content, and take full responsibility for the content of this publication). The device was turned on and the prescribed cycle was run according to the manufacturer’s protocol. To ensure the Holder pasteurization conditions, an external thermal probe was put inside the PET container (temperature curves are presented on Figure S2). Test tubes with non-treated virus + breast milk mixes were held in the fridge for the duration of the pasteurization cycle. Afterwards, non-treated and treated mixes were immediately titrated in cell culture to determine infectious virus titer, and stored at −70 °C for viral genome detection via RT-PCR or PCR.

Each virus + breast milk mix was prepared separately, then divided in two test tubes: one treated, one non-treated—each used for infectious virus titration or RT-PCR/PCR investigation. Therefore, experiments for each virus in each breast milk sample were independently repeated 3–9 times (Tables S3 and S4).

### 2.5. Infectious Virus Detection and Titration in Cell Culture (CCID_50_)

Consequential 10-fold dilutions of the virus-containing sample were prepared in a cultural medium appropriate for the cell line (Table 2 and Supplementary Material) and then added to the cell monolayers (Vero cells) or mixed with cell suspensions (3 × 10^4^ cells per well; for MT4, RD, and Hep2c cells) on 96-well plates. Cells were incubated at 37 °C, 5% CO_2_ for 10 (MT4), 7 (Hep2c), and 5 (RD, Vero) days. Then, virus-induced cytopathic effect (cell death) signs were detected via microscope. Virus titers were calculated with the Karber formula [42] and expressed as 50% Cell Culture Infectious Dose (CCID_50_) per mL.

Due to the cytotoxicity of breast milk, minimal dilution in the virus titration was 10^−1^. Therefore, the sensitivity threshold (detection limit) in experiments was 1.625 log CCID_50_/mL.

Virus titer reduction was calculated as the difference between titers of non-treated and treated tubes. In cases where an infectious virus was not detected, the sensitivity threshold was used to calculate virus titer reduction.

### 2.6. RT-PCR and PCR

Viral nucleic acid was isolated from virus + breast milk mixtures using RIBOPrep (AmpliSense, Moscow, Russia), according to the manufacturer’s instructions.

RT-PCR (HIV-1, CHIKV, SARS-CoV-2, PV1) and PCR (hAdV5) were performed with qScript Virus 1-Step RT-qPCR ToughMix (QuantaBio, Beverly, MA, USA) and specific sets of primers [43,44,45,46,47] (Table S2). Treated and non-treated samples were analyzed in the same PCR run.

Virus genome content reduction was calculated as the difference between the Ct values of the non-treated and treated tubes: 2^ΔCt^ = 2^(Ct (non-treated) − Ct (treated)^.

### 2.7. Statistical Analysis

Experimental data were processed using descriptive statistics, calculating geometric mean (mean) and standard deviation (SD). Statistical differences between virus titers and viral genome contents of treated and non-treated samples were assessed using the Mann–Whitney test. Statistical calculations and visualizations were performed using OriginPro 8 (OriginLab Corp., Northampton, MA, USA).

## 3. Results

To determine the efficacy of Holder pasteurization with the portable pasteurizer, viruses with different virions that can be found in breast milk and/or are considered a threat to the newborn during breastfeeding (HIV-1, SARS-CoV-2, CHIKV, RSV, enteroviruses, hAdV, and HSV-1) were chosen (Table 2).

### 3.1. Infectious Virus Decrease After Holder Pasteurization of Virus-Contaminated Breast Milk

**Figure 1:**
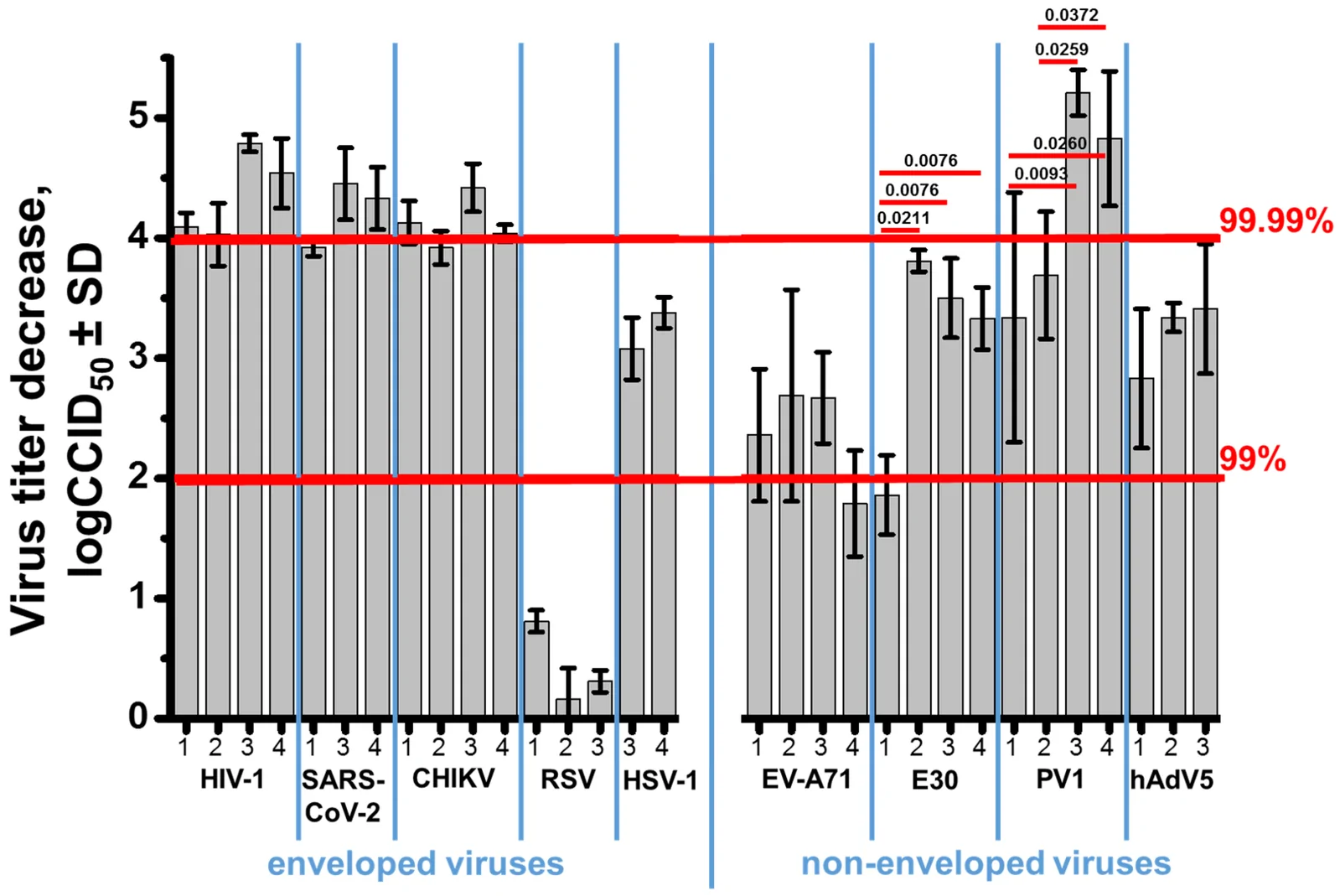
Infectious virus titer decrease (log CCID_50_) after Holder pasteurization of virus-contaminated breast milk in the portable pasteurizer. Data (virus titer decrease) for each breast milk sample are presented as mean ± SD. The breast milk sample (1–4) used in each experiment is signified as a number below the X-axis. The number of replicates (N) for each virus with each breast milk sample is three–nine. Comparisons are made with the Mann–Whitney test; significant differences (*p* < 0.05) are shown above the histogram. Red lines show 2 log CCID_50_ (or 99%) and 4 log CCID_50_ (or 99.99%) virus titer reduction.

Holder pasteurization of breast milk containing 5.5–6.5 log CCID_50_ of enveloped RNA viruses HIV-1 (family *Retroviridae*), SARS-CoV-2 (family *Coronaviridae*), and CHIKV (family *Togaviridae*) led to a decrease in virus infectivity below the detection limit in tested breast milk samples, resulting in at least 4 log CCID_50_ (or 99.99%) reduction in infectivity (Figure 1, Table S3).

However, another enveloped RNA virus, RSV (family *Pneumoviridae*), showed the lowest susceptibility to pasteurization with only 0.16–0.81 log CCID_50_ infectivity reduction (Figure 1, Table S3).

Human RNA viruses with non-enveloped stable capsids (Family *Picornaviridae*), representing three *Enterovirus* species (EV-A71, E30, and PV1), showed varied infectivity reduction by pasteurization. EV-A71 was inactivated quite effectively in all four breast milk samples with an average infectivity reduction of 2.35 ± 0.53 log CCID_50_, i.e., more than 99%. E30 was also inactivated quite effectively with mean virus titer decrease of 2.64 ± 0.90 log CCID_50_; however, the inactivation efficiency in sample 1 was significantly lower than in samples 2–4. PV1 was inactivated, on average, more effectively than other enteroviruses with a virus titer reduction of 4.01 ± 1.06 log CCID_50_, i.e., more than 99.99%. Moreover, a statistically significant difference in virus titer decrease was observed between breast milk samples 1–2 and 3–4 (Figure 1, Table S3).

Holder pasteurization of breast milk samples 3 and 4 contaminated with enveloped DNA virus HSV-1 (family *Orthoherpesviridae*) resulted in a significant reduction in infectious virus titer by 3.22 ± 0.24 log CCID_50_. It is worth noting that with virtually the same virus titer in non-treated sample in breast milk 3 the virus was not inactivated below the detection limit, and in sample 4 the infectious titer decreased to undetectable levels (Figure 1, Table S3).

Non-enveloped DNA virus hAdV5 (family *Adenoviridae*) lost 3.13 ± 0.53 log CCID_50_ (more than 99.9%) infectivity after pasteurization with one–three breast milk samples (Figure 1, Table S3).

### 3.2. Genome Nucleic Acid Decrease

**Figure 2:**
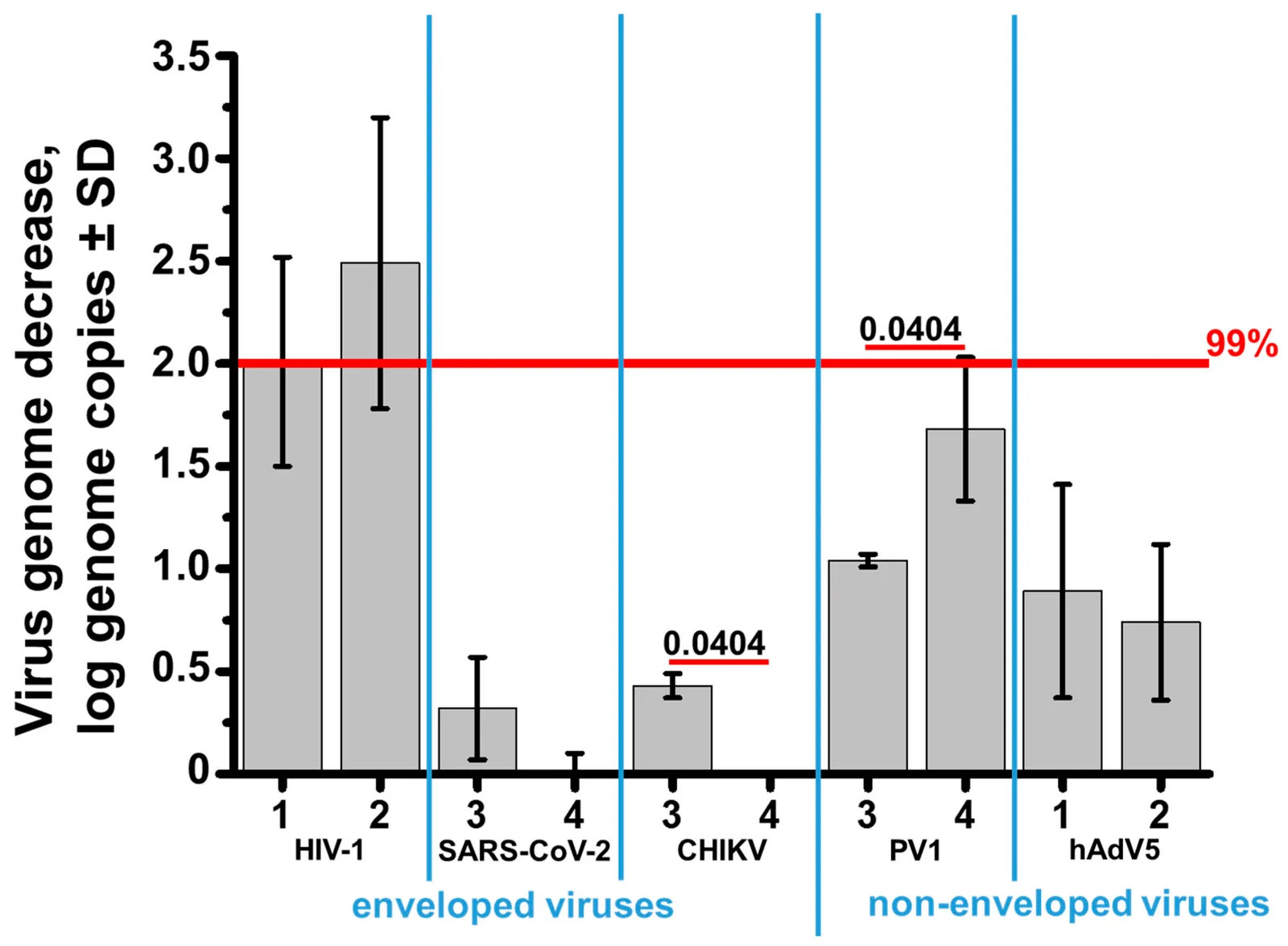
Viral genome content reduction after Holder pasteurization of virus-contaminated breast milk in the portable pasteurizer. Data for each breast milk sample are presented as mean ± SD. The breast milk sample (1–4) used in each experiment is signified as a number below the X-axis. The number of replicates (N) for each virus with each breast milk sample is three–six. Comparisons are made with the Mann–Whitney test; significant differences (*p* < 0.05) are shown above the histogram. Red lines show 2 log (or 99%) reduction.

The reduction in viral genome content for enveloped RNA viruses (HIV-1, CHIKV, and SARS-CoV-2), which demonstrated the most significant reduction in infectivity (below the detection limit), was less pronounced (Figure 2, Table S4). HIV-1 genome content was reduced by more than 2 log genome copies (or 99%), but for CHIKV and SARS-CoV-2 the reduction was less than 0.5 logs.

The reduction in genome content for non-enveloped RNA virus PV1 was 1.36 ± 0.42 log genome copies. The reduction in genome content for stable DNA virus hAdV5 was less than 1 log genome copies (Figure 2, Table S4).

## 4. Discussion

Breast milk is optimal for newborn feeding for the first 6 months of life, as it includes not only nutrition but provides a child with antibodies, microbiota, etc. [1,2,3,4]. However, breastfeeding can be a source of infection for a newborn [6,7,8,10,11,12,13,14,15,16,17,18,19,20,21,22,23,24,25,26,27,28]. There are a number of recommendations for physicians as to how to address these cases, although not all of the situations are covered [9].

Holder pasteurization is a gold-standard approach for the antiviral treatment of breast milk. Previously, the efficiency of Holder pasteurization has been studied against a large variety of viruses, but in different experimental settings: different devices, milk or non-milk matrices [40]. These data were extrapolated to the related viruses and used as a basis for the efficiency of the breast milk pasteurizers using the Holder pasteurization method. However, infectious virus survival during pasteurization can be determined by several factors, including virion structure and milk properties (fat content, bio-active compounds, etc.), both of which can vary among closely related species, and therefore cannot be extrapolated correctly.

The main aim of the present work was to study the virucidal activity of Holder pas-teurization against RNA and DNA viruses with different virion compositions in breast milk samples from different donors in a controlled experimental setting in a portable pas-teurizer. Viruses were selected on the basis of their ability to infect humans, contaminate breast milk during acute infection of a mother (HIV-1, CHIKV, SARS-CoV-2, enteroviruses, and RSV), or infect newborns during breastfeeding as well as contaminate milk during expression (HSV-1, hAdV5). Moreover, we have studied not only infectious virus reduction, but also genome content reduction to probably shed some light on the mechanism of the infectivity decrease.

Three enveloped RNA viruses (HIV-1, SARS-CoV-2, and CHKV) were the most susceptible to Holder pasteurization. Decrease in infectious virus titer for these viruses has been over 4 log CCID_50_ (or 99.99%), which is considered high virucidal efficiency according to the national guidelines [48]. The results for HIV-1 and SARS-CoV-2 correspond with previously obtained data [31,49], while for CHIKV in breast milk this is new data. However, other alphaviruses have been shown to be inactivated in breast milk by Holder pasteurization (4.2 log PFU/mL reduction, [40]). In recent years, CHIKV has spread in Southern Europe and China and caused outbreaks [50]. This creates a possible threat for newborns [51] that can be partially mitigated by Holder pasteurization of breast milk.

Another studied enveloped RNA virus was RSV, a known threat to newborns. Unfortunately, even in comparatively low doses, RSV was the least susceptible to inactivation via pasteurization. No similar data have been found in the literature, suggesting that this is the first evaluation of the effectiveness of Holder pasteurization for RSV in breast milk.

Three species of *Enterovirus* genus that infect humans were tested in the present work as non-enveloped RNA viruses. It is worth noting that the selected strains of EV-A71 (*E. alhacoxsackie*) and E30 (*E. betacoxsackie*) have been associated with outbreaks of diseases with CNS damage and have a predominantly oral–fecal route of transmission [52,53]. In addition, PV1 (*E. coxsackiepol*) vaccine strain Sabin1 is also characterized by the same route of transmission. All three viruses were inactivated by Holder pasteurization, although infectivity reduction in these enteroviruses during treatment significantly differed in the series EV-A71 (about 2 log CCID_50_) < E30 < PV1 (about 4 log CCID_50_). Overall, these data are consistent with published data for CVB4 (*E. betacoxsackie*) and PV1 [54,55], although PV1 in breast milk was tested for the first time. We can conclude that viruses belonging to one genus can differ in stability during pasteurization. Therefore, if a new virus variant or a new virus emerges, it most likely should be subjected to testing for Holder pasteurization efficiency.

We tested two DNA viruses: non-enveloped hAdV5 and enveloped HSV-1. Both viruses showed over 3 log CCID_50_ decrease in infectivity after Holder pasteurization in breast milk in a portable device. Similar results are described in the literature [54,56].

Infectivity reduction in E30 and PV1 in different samples of breast milk significantly differed. The lowest reduction was observed in sample 1; the same tendency was observed with other viruses but the differences were not statistically significant. Although we did not study the content of breast milk samples, we can suggest that breast milk properties can affect the efficiency of Holder pasteurization.

Besides assessing the infectivity reduction, we have studied the genome content de-crease during Holder pasteurization using PCR. The results showed a significant, but low, decrease in HIV-1 genomic RNA and hAdV5 genomic DNA content. Considering that PCR detects very short fragments of the viral genome, the lack of Ct change after pasteurization does not allow one to confirm the integrity of the genomic molecule.

Outbreaks of emerging and re-emerging diseases can occur unexpectedly. In the COVID-19 pandemic a huge burden on neonatal facilities was caused by the absence of guidelines and tested approaches on how to proceed with newborns from SARS-CoV-2-infected mothers [57]. Therefore, Holder pasteurization can be a tool in a novel pandemic preparedness toolbox.

## Figures and Tables

**Table 1:** Viruses found in breast milk and risk of transmission during breastfeeding.

Genome	Virion	Virus Family	Virus (Common Name)	Transmission Likelihood	Reference
DNA	Enveloped	Orthoherpesviridae	CMV	High	[10]
			VZV	Low	[11]
			EBV	Low	[12]
			HHV-8	Low	[13]
		Orthopoxviridae	Mpox	Low	[14]
	Non-enveloped	Papillomaviridae	HPV	Low	[15]
+RNA	Enveloped	Hepaciviridae	HCV	Low	[16]
		Flaviviridae	YFV	High	[17,18]
			ZIKV	Unknown	[8]
			DENV	Unknown	[19]
			WNV	Low	[20]
			TBEV	Low	[21]
		Togaviridae	CHIKV	Unknown	[22]
			SINV, SFV	Low	[23]
		Coronaviridae	SARS-CoV-2	Low	[24]
	Non-enveloped	Hepeviridae	HEV	Low	[25]
−RNA	Enveloped	Filoviridae	EBOV	High	[26]
		Hantaviridae	Andes	High	[27]
RNA with RT	Enveloped	Retroviridae	HTLV	High	[7]
			HIV	High	[6]
DNA with RT	Enveloped	Hepadnaviridae	HBV	Low	[28]

**Table 2:** Virus strains and cell lines used in the study.

Virus Taxonomy (Family, Genus, Species Names)	Genome	Virion	Virus Common Name (Abbreviation)	Strain	ID (Catalog Number or Sequence Database ID)	Cell Line
Family *Retroviridae* Genus *Lentivirus* *Lentivirus humimdef1*	+RNA	Enveloped	Human immunodeficiency virus 1 (HIV-1)	NL4-3	MHRA cat. #ARP2006	MT4
Family *Togaviridae* Genus *Alphavirus* *Alphavirus chikungunya*	+RNA	Enveloped	Chikungunya virus (CHIKV)	Nic	GenBank ID MN271691-2	Vero
Family *Coronaviridae* Genus *Betacoronavirus* *Betacoronavirus pandemicum*	+RNA	Enveloped	SARS-CoV-2	10969	GisAid ID EPI_ISL_18525720	Vero
Family *Picornaviridae* Genus *Enterovirus* *Enterovirus alphacoxsackie*	+RNA	Non-enveloped	Enterovirus A71 (EV-A71)	46973	GenBank ID KJ645808	RD
Family *Picornaviridae* Genus *Enterovirus* *Enterovirus betacoxsackie*	+RNA	Non-enveloped	Echovirus 30 (E30)	48461	GenBank ID MK704489	RD
Family *Picornaviridae* Genus *Enterovirus* *Enterovirus coxsackiepol*	+RNA	Non-enveloped	Poliovirus type 1 (PV1)	Sabin1	GenBank ID AY184219	RD
Family *Pneumoviridae* Genus *Orthopneumovirus* *Orthopneumovirus hominis*	−RNA	Enveloped	Respiratory syncytial virus (RSV)	RSV long	ATCC cat. #VR-26 GenBank ID MW039343	Vero
Family *Adenoviridae* Genus *Mastadenovirus* *Mastadenovirus caesari*	dsDNA	Non-enveloped	Human adenovirus 5 (hAdV5)	14286	–	Hep2c
Family *Orthoherpesviridae* Genus *Simplexvirus* *Simplexvirus humanalpha 1*	dsDNA	Enveloped	Herpes simplex virus type 1 (HSV-1)	IDoG	GKV (Russia) cat. #3035	Vero

+RNA—positive single-stranded RNA. −RNA—negative single-stranded RNA. dsDNA—double-stranded DNA. Enveloped—virion contains a lipid envelope.

## Data Availability

All data are presented within the manuscript and Supplementary Materials file.

## Supplementary Materials

The following supporting information can be downloaded at: https://www.mdpi.com/article/10.3390/pathogens15091000/s1, Figure S1: Experimental setup scheme and design. (A) A raft with test tubes containing breast milk; (B) a PET-container with cowʹs milk (290 mL) and a raft with test tubes; (C) a bottle holder inside the pasteurizer water bath; (D) the pasteurizer with a lid (and temperature probe) (images generated from photographs using Samsung AI); and (E) schematic view (not to scale) of the test tube with contaminated breast milk in the pasteurizer; Figure S2: Temperature curves for the heating element (red), water (blue), and milk in a PET container (gray) during Holder pasteurization (initial water and milk temperatures were room temperature). The pasteurization cycle (30 min) is highlighted in yellow; the mean temperature ± standard deviation in the milk in the experimental PET container during pasteurization is shown as numbers; Table S1: Demographic characteristics of breast milk donors; Table S2: RT-PCR and PCR primers and probes; Table S3: Raw infectious virus titers in treated and non-treated breast milk samples; Table S4: Raw Ct values in treated and non-treated breast milk samples. References [43,44,45,46,47] are cited in the supplementary materials.

## Abbreviations

The following abbreviations are used in this manuscript:

AAP	The American Academy of Pediatrics
CCID_50_	s 50% Cell Culture Infectious Dose
CHIKV	Chikungunya virus
CMV	Cytomegalovirus
DENV	Dengue virus
E30	Echovirus 30
EBOV	Ebola virus
EBV	Epstein-Barr virus
EV-A71	Enterovirus A71
hAdV5	Human adenovirus 5
HCV	Hepatitis virus C
Hep2c	Epidermoid carcinoma cell line
HEV	Hepatitis virus E
HHV-8	Human herpesvirus 8
HIV-1	Human immunodeficiency virus
HPV	Human papillomavirus
HSV-1	Herpes simplex virus 1
Mpox	Monkey orthopoxvirus
MT4	Human leukemia T-cell line
PCR	Polymerase chain reaction
PET	Polyethylene
PV1	Poliovirus 1
RD	Rhabdomyosarcoma cell line
RSV	Respiratory syncytial virus
RT-PCR	Reverse transcription-Polymerase chain reaction
SARS-CoV-2	Severe acute respiratory syndrome coronavirus 2
SFV	Semliki forest virus
SINV	Sindbis virus
TBEV	Tick-borne encephalitis virus
Vero	Green monkey kidney cell line
VZV	Varicella-Zoster virus
WNV	West Nile virus
YFV	Yellow fever virus
ZIKV	Zika virus
