# Supplementary material for: Virucidal Efficacy of Holder Pasteurization of Breast Milk in a Portable Device
Source: Pathogens. 2026 Sep 21;15(9):1000. doi: 10.3390/pathogens15091000 (PMC13610739; doi:10.3390/pathogens15091000)
Supplement: Supplementary file 1 [file pathogens-15-01000-s001.zip › pathogens-4538071-supplementary.pdf]

Supplementary material

Virucidal efficacy of Holder pasteurization of breast milk in a portable device

Maxim I. Suchkov[1,2], Elizaveta V. Yakovchuk[1], Elena Y. Shustova[1], Dina I. Sirazova[1], Anastasia D. Zolotareva[1], Evgenia V. Karpova[1], Ekaterina L. Aksenova[3], Svetlana E. Sotskova[1], and Liubov I. Kozlovskaya[1,2*]

[1] Chumakov Federal Scientific center for Research-and-Development of Immune-and-Biological Preparations of RAS (Institute of poliomyelitis), Moscow, Russia;
[2] Sechenov First Moscow State Medical University (Sechenov University), Moscow, Russia;
[3] Biobank, Institute of regenerative medicine, First Moscow State Medical University (Sechenov University), Moscow, Russia
* Correspondence: kozlovskaya_li@chumakovs.su, +7(495)841-9054

1. Materials and Methods

1.1. Cells and viruses

Cell lines used in a present study:

- Vero green monkey kidneys (WHO Biologicals, Switzerland);
- RD human rhabdomyosarcoma (MHRA, UK);
- Hep2c epidermoid carcinoma (MHRA, UK);
- MT4 – T lymphocytes infected with primate T-cell leukemia virus type 1 (PTLV-1) (MHRA, UK).

The cells were passaged in a medium appropriate for a particular cell line: for Vero cells – DMEM (Chumakov FSC R&D IBP RAS (Institute of poliomyelitis), Russia) with 5% fetal bovine serum (FBS, Invitrogen, South America) and penicillin, streptomycin (PanEco, Russia); for RD and Hep2c cells – Eagle MEM with a doubled of amino acids and vitamins (Chumakov FSC R&D IBP RAS (Institute of poliomyelitis), Russia) with 5% FBS (Invitrogen, South America) and penicillin, streptomycin (PanEco, Russia); for MT4 cells – RPMI-1640 (Chumakov FSC R&D IBP RAS (Institute of poliomyelitis), Russia) with 10% FBS (Invitrogen, South America) and gentamicin (PanEco, Russia).

Viruses were received from ATCC (USA), MHRA (UK), National virus collection (GKV, Russia), or from Chumakov FSC R&D IBP RAS (Institute of poliomyelitis) virus collection. For the study viruses were multiplied in appropriated cells, aliquoted and stored at -70 °C.

1.2. Breast milk donors

Table S1. Demographic characteristics of breast milk donors.

Sample	Age, years	Pregnancy	Time after giving birth, months
1	35	2	0
2	33	3	6
3	34	2	3
4	32	2	1

1.3. Experimental setup scheme and design

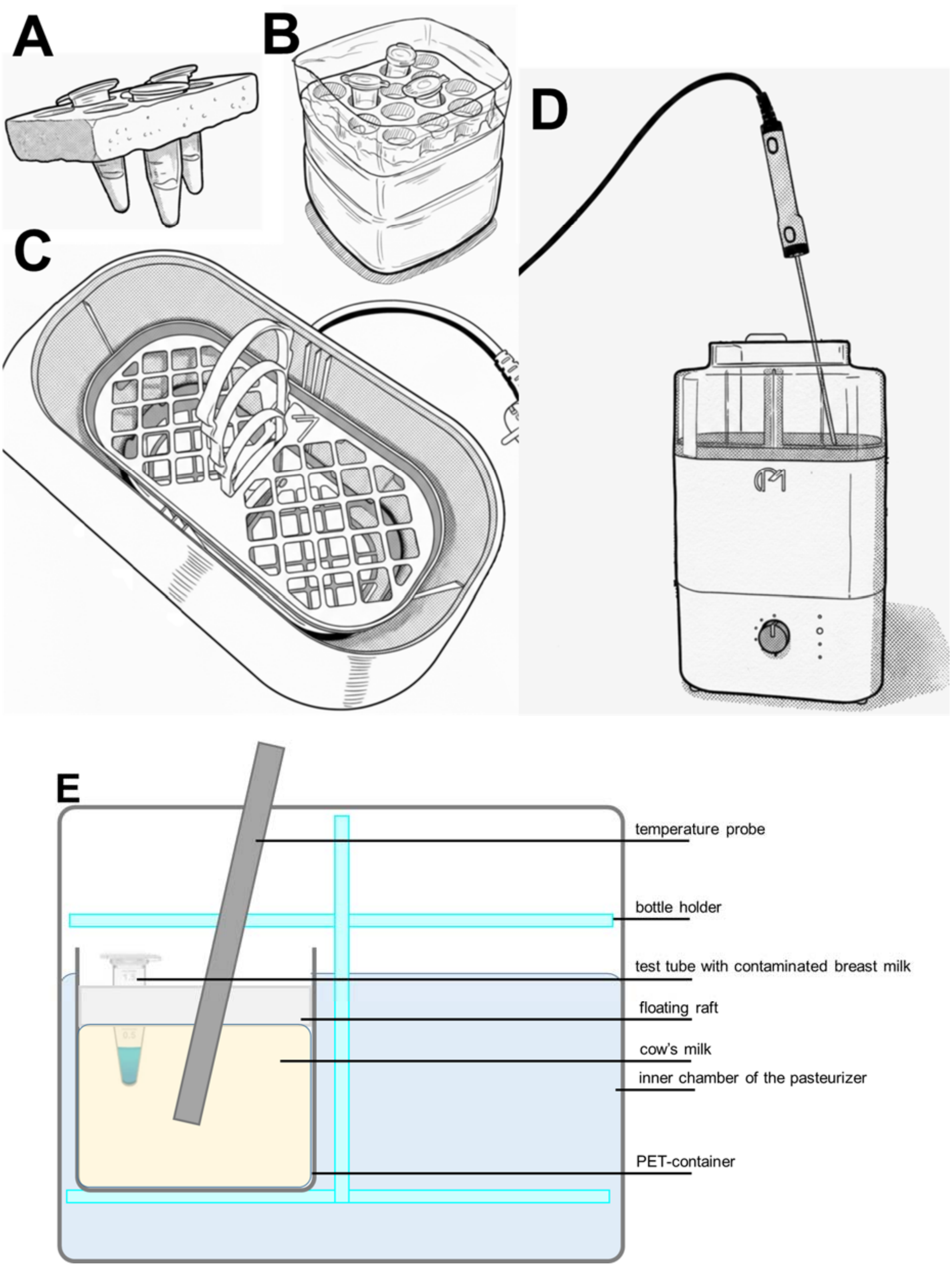

Figure S1. Experimental setup scheme and design. A) A raft with test tubes containing breast milk; B) A PET-container with cow's milk (290 ml) and a raft with test tubes; C) A bottle holder inside the pasteurizer water bath; D) The pasteurizer with a lid (and temperature probe) (images generated from photographs using Samsung AI); E) Schematic view (not to scale) of the test tube with contaminated breast milk in the pasteurizer.

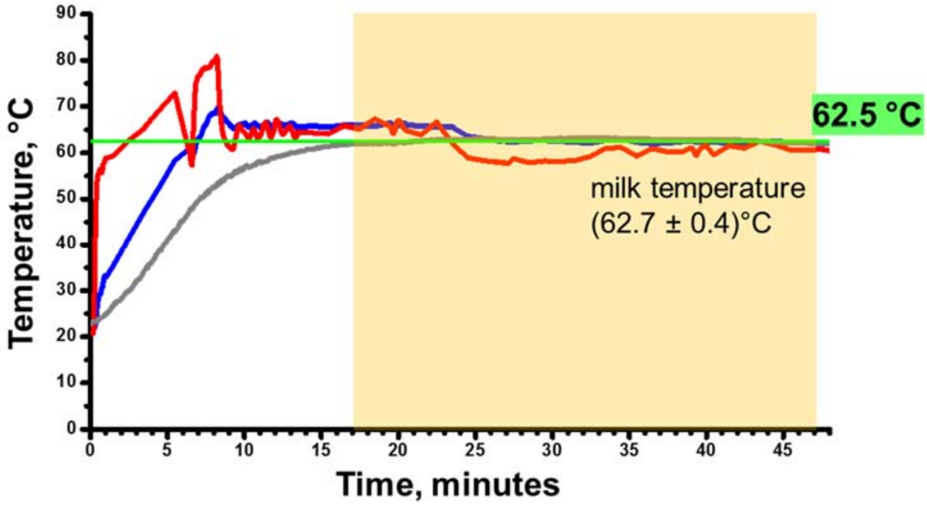

Figure S2. Temperature curves for the heating element (red), water (blue), and milk in a PET-container (gray) during Holder pasteurization (initial water and milk temperatures were room temperature). The pasteurization cycle (30 min) is highlighted in yellow; the mean temperature ± standard deviation in the milk in the experimental PET-container during pasteurization is shown as numbers.

1.4. RT-PCR and PCR primers and probes

Table S2. RT-PCR and PCR primers and probes

Virus	Forward primer	Reverse primer	Taq-man probe	Reference
HIV-1 (genomic RNA)	P9501 CAGATGCTGCATATAAGCAGCTG	10T20 TTTTTTTTTTTTTTTTTTTTTTGAAGCACTC	ProbeNL4-3 FAM-CCTG-TACTGGGTCTCTCTGG-BHQ-1	[43]
SARS-CoV-2	E_F1 ACAGGTACGTTAA-TAGTTAATAGCGT	E_R2 ATATTGCAGCAG-TACGCACACA	E_P1 FAM-ACAC-TAGCCATCCTTACTGCGCTTCG-BBQ	[44]
CHIKV	F-CHIK AA-GCTYCGCGTCCTTTAC-CAAG	R-CHIK CCAAATT-GTCCYGGTCTTCCT	P-CHIK FAM-CCAATGT-CYTCMGCCTGGACACCTTT-BHQ1	[45]
PV1	S1-A CCACTGGCTTCAG-TGTTT	S1-S AGGTCAGATGCTT-GAAAGC	S1 Probe CY5-CGCCCCCACCGTTTCAC-GGA-BHQ3	[46]
hAdV5	Adeno-8703F CGACGAC-GCGICGRTTGA	Adeno-8873R CGAGATCGCCTAT-CARGAYAACTC	Adeno-8771Pr ROX-GAACCTGAAAGANAG-TTCRACAGAATCAAT-BHQ2	[47]

2. Results

2.1. Virus titration – raw data

Table S3. Raw infectious virus titers in treated and non-treated breast milk samples

Virus	Breast milk sample	Virus titer, log $CCID_{50}$/ml		Titer decrease, $logCCID_{50}$	Mean virus titer decrease for each breast milk sample, $logCCID_{50}$ ± SD	Mean virus titer decrease for each virus, $logCCID_{50}$ ± SD
		non-treated	treated			
HIV-1	1	5.875	<1.625*	4.250	4.09 ± 0.12	4.32 ± 0.37
		5.625	<1.625	4.0		
		5.625	<1.625	4.0		
		5.750	<1.625	4.125		
	2	5.875	<1.625	4.250	4.03 ± 0.26	
		5.500	<1.625	3.875		
		5.375	<1.625	3.750		
		5.875	<1.625	4.250		
	3	6.50	<1.625	4.875	4.79 ± 0.07	
		6.375	<1.625	4.750		
		6.375	<1.625	4.750		
	4	6.0	<1.625	4.375	4.54 ± 0.29	
		6.50	<1.625	4.875		
		6.0	<1.625	4.375		
SARS-CoV-2	1	5.50	<1.625	3.875	3.92 ± 0.07	4.24 ± 0.32
		5.50	<1.625	3.875		
		5.625	<1.625	4.0		
	3	5.750	<1.625	4.125	4.45 ± 0.31	
		6.125	<1.625	4.50		
		6.375	<1.625	4.750		
	4	6.250	<1.625	4.625	4.33 ± 0.26	
		5.750	<1.625	4.125		
		5.875	<1.625	4.250		
CHIKV	1	5.625	<1.625	4.0	4.13 ± 0.18	4.13 ± 0.22
		5.750	<1.625	4.125		
		5.625	<1.625	4.0		
		6.0	<1.625	4.375		
	2	5.625	<1.625	4.0	3.92 ± 0.14	
		5.625	<1.625	4.0		
		5.375	<1.625	3.750		
		5.750	<1.625	4.125		
	3	6.250	<1.625	4.625	4.42 ± 0.20	
		5.875	<1.625	4.25		
		6.00	<1.625	4.375		
	4	5.75	<1.625	4.125	4.04 ± 0.07	
		5.625	<1.625	4.0		
		5.625	<1.625	4.0		
EV-A71	1	5.875	3.50	2.375	2.36 ± 0.55	2.35 ± 0.53
		6.125	2.625	3.50		
		5.375	3.0	2.375		

Virus	Breast milk sample	Virus titer, log CCID50/ml		Titer decrease, logCCID50	Mean virus titer decrease for each breast milk sample, logCCID50 ± SD	Mean virus titer decrease for each virus, logCCID50 ± SD
		non-treated	treated			
		6.250	4.0	2.250		
		6.125	3.875	2.250		
		5.625	3.875	1.750		
		6.0	4.0	2.0		
	2	4.250	1.625	2.625	2.69 ± 0.88	
		4.375	1.625	2.750		
	3	7.125	4.125	3.0	2.67 ± 0.38	
		7.250	5.0	2.250		
		7.125	4.375	2.75		
	4	7.0	5.625	1.375	1.79 ± 0.44	
		7.375	5.625	1.750		
		7.250	5.0	2.250		
E30	1	5.625	3.250	2.375	1.86 ± 0.33	2.64 ± 0.90
		5.625	3.875	1.750		
		5.875	4.50	1.375		
		6.0	4.250	1.750		
		5.750	3.375	2.375		
		5.750	4.0	1.750		
		5.875	4.250	1.625		
		6.0	4.125	1.875		
		5.875	4.0	1.875		
	2	5.50	<1.625	3.875	3.81 ± 0.09	
		5.375	<1.625	3.750		
	3	5.50	<1.625	3.875	3.50 ± 0.33	
		5.0	<1.625	3.375		
		4.875	1.625	3.250		
	4	5.250	<1.625	3.625	3.33 ± 0.26	
		4.750	<1.625	3.125		
		4.875	<1.625	3.250		
PV1	1	6.750	4.50	2.250	3.34 ± 1.04	4.01 ± 1.06
		6.0	<1.625	4.375		
		5.625	<1.625	4.0		
		6.250	<1.625	4.625		
		6.625	3.25	3.375		
		6.750	4.0	2.750		
		6.250	4.250	2.0		
		6.750	4.50	2.25		
	2	5.250	<1.625	3.625	3.69 ± 0.53	
		4.625	<1.625	3.0		
		5.50	<1.625	3.875		
		5.875	<1.625	4.250		
	3	6.750	1.750	5.0	5.21 ± 0.19	
		7.0	1.750	5.250		
		7.375	2.0	5.375		
	4	6.0	1.75	4.250	4.83 ± 0.56	

Virus	Breast milk sample	Virus titer, log $CCID_{50}$/ml		Titer decrease, log$CCID_{50}$	Mean virus titer decrease for each breast milk sample, log$CCID_{50}$ ± SD	Mean virus titer decrease for each virus, log$CCID_{50}$ ± SD
		non-treated	treated			
		7.250	1.875	5.375		
		7.0	2.125	4.875		
RSV	1	2.50	1.625	0.875	0.81 ± 0.09	0.35 ± 0.33
		2.50	1.75	0.750		
	2	2.50	2.50	0.0	0.16 ± 0.24	
		2.50	2.375	0.125		
		2.50	2.0	0.50		
		2.50	2.50	0.0		
	3	3.875	3.625	0.250	0.31 ± 0.09	
		3.875	3.50	0.375		
HSV-1	3	5.375	2.0	3.375	3.08 ± 0.26	3.22 ± 0.24
		5.50	2.50	3.0		
		5.375	2.50	2.875		
	4	5.0	<1.625	3.375	3.38 ± 0.13	
		5.125	<1.625	3.50		
		4.875	<1.625	3.250		
hAdV5	1	7.250	4.375	2.875	2.83 ± 0.58	3.13 ± 0.53
		7.125	4.250	2.875		
		6.250	4.125	2.125		
		6.50	4.50	2.0		
		7.375	4.125	3.250		
		7.50	4.25	3.250		
		7.250	3.75	3.50		
	2	7.500	4.125	3.375	3.34 ± 0.12	
		7.375	4.125	3.25		
		7.500	4.250	3.25		
		7.250	3.750	3.50		
	3	4.625	<1.625	3.0	3.41 ± 0.54	
		4.50	<1.625	2.875		
		5.50	<1.625	3.875		
		5.50	1.625	3.875		

<1.625 – below detection limit

2.2. Virus genome decrease – raw data

Table S4. Raw Ct values in treated and non-treated breast milk samples

Virus	Breast milk sample	Ct, cycle		Decrease, log genome copies	Mean genome decrease for breast milk sample, log genome copies ± SD	Mean genome decrease for virus, log genome copies ± SD
		non-treated	treated			
HIV-1	1	27.04	31.14	1.23422	2.01 ± 0.51	2.23 ± 0.63
		28.82	35.85	2.11624		
		25.98	31.61	1.6948		
		26.46	32.83	1.91756		
		28.56	37.3	2.631		
		25.64	33.85	2.47146		
	2	25.47	33.98	2.56177	2.49 ± 0.71	
		27.78	35.82	2.42028		
		26.36	31.13	1.43591		
		29.2	37.8	2.58886		
		25.71	37.07	3.4197		
SARS-CoV-2	3	10.49	10.8	0.09332	0.32 ± 0.25	0.17 ± 0.24
		10.51	11.39	0.26491		
		10.67	12.63	0.59002		
	4	10.40	10.78	0.11439	0.01 ± 0.09	
		10.19	10.04	-0.04515		
		10.01	9.92	-0.02709		
CHIKV	3	17.34	18.58	0.37328	0.43 ± 0.06	-0.15 ± 0.64
		17.33	18.77	0.43348		
		17.31	18.96	0.4967		
	4	19.40	16.94	-0.74053	-0.74 ± 0.01	
		19.35	16.85	-0.75257		
		19.15	16.73	-0.72849		
PV1	3	11.79	15.35	1.07167	1.04 ± 0.03	1.36 ± 0.42
		12.07	15.48	1.02651		
		12.06	15.42	1.01146		
	4	11.94	18.3	1.91455	1.68 ± 0.35	
		11.63	17.77	1.84832		
		14.30	18.52	1.27035		
hAdV5	1	16.24	20.04	1.14391	0.89 ± 0.52	0.81 ± 0.43
		16.05	21.04	1.50214		
		16.54	18.22	0.50573		
		17.04	18.41	0.41241		
	2	16.17	19.57	1.0235	0.74 ± 0.38	
		16.05	19.71	1.10177		
		16.43	17.52	0.32812		
		17.38	19.02	0.49369		

References

43. Shan L, Rabi SA, Laird GM, Eisele EE, Zhang H, Margolick JB, Siliciano RF. A novel PCR assay for quantification of HIV-1 RNA. J Virol. 2013;87(11):6521-5. doi: 10.1128/JVI.00006-13.
44. Ramírez JD, Muñoz M, Hernández C, Flórez C, Gomez S, Rico A, Pardo L, Barros EC, Paniz-Mondolfi AE. Genetic Diversity Among SARS-CoV2 Strains in South America may Impact Performance of Molecular Detection. Pathogens. 2020;9(7):580. doi: 10.3390/pathogens9070580.
45. Pastorino B, Bessaud M, Grandadam M, Murri S, Tolou HJ, Peyrefitte CN. Development of a TaqMan RT-PCR assay without RNA extraction step for the detection and quantification of African Chikungunya viruses. J Virol Methods. 2005;124(1-2):65-71. doi:10.1016/j.jviromet.2004.11.002
46. Gerloff N, Sun H, Mandelbaum M, Maher C, Nix WA, Zaidi S, Shaukat S, Seakamela L, Nalavade UP, Sharma DK, Oberste MS, Vega E. Diagnostic Assay Development for Poliovirus Eradication. J Clin Microbiol. 2018;56(2):e01624-17. doi: 10.1128/JCM.01624-17.
47. Osolodkin DI, Kozlovskaya LI, Iusupov IR, Kurkin AV, Shustova EY, Orlov AA, Khvatov EV, Mutnykh ES, Kurashova SS, Vetrova AN, Yatsenko DO, Goryashchenko AS, Ivanov VN, Lukyanenko ER, Karpova EV, Stepanova DA, Volok VP, Sotskova SE, Dzagurova TK, Karganova GG, Lukashev AN, Ishmukhametov AA. Phenotypic assessment of antiviral activity for spiro-annulated oxepanes and azepenes. Chem Biol Drug Des. 2024;103(5):e14553. doi: 10.1111/cbdd.14553.
